# Controlling the Adhesion of Superhydrophobic Surfaces Using Electrolyte Jet Machining Techniques

**DOI:** 10.1038/srep23985

**Published:** 2016-04-05

**Authors:** Xiaolong Yang, Xin Liu, Yao Lu, Shining Zhou, Mingqian Gao, Jinlong Song, Wenji Xu

**Affiliations:** 1 Key Laboratory for Precision and Non-traditional Machining Technology of the Ministry of Education, Dalian University of Technology, Dalian 116023, People’s Republic of China; 2 Department of Chemistry, University College London, 20 Gordon Street, London, WC1H 0AJ, UK

## Abstract

Patterns with controllable adhesion on superhydrophobic areas have various biomedical and chemical applications. Electrolyte jet machining technique (EJM), an electrochemical machining method, was firstly exploited in constructing dimples with various profiles on the superhydrophobic Al alloy surface using different processing parameters. Sliding angles of water droplets on those dimples firstly increased and then stabilized at a certain value with the increase of the processing time or the applied voltages of the EJM, indicating that surfaces with different adhesion force could be obtained by regulating the processing parameters. The contact angle hysteresis and the adhesion force that restricts the droplet from sliding off were investigated through experiments. The results show that the adhesion force could be well described using the classical Furmidge equation. On account of this controllable adhesion force, water droplets could either be firmly pinned to the surface, forming various patterns or slide off at designed tilting angles at specified positions on a superhydrophobic surface. Such dimples on superhydrophopbic surfaces can be applied in water harvesting, biochemical analysis and lab-on-chip devices.

Superhydrophobic surface, which has a water contact angle (CA) larger than 150°, is an ideal water-repellent surface due to the property of ultralow water affinity. Water droplets on the superhydrophobic surface can easily slide off when the surface is slightly tilted. This kind of surface has a large application prospect in the fields of self-cleaning^1^, drag reduction^2^, anti-icing^3^ and oil/water separation^4^,^5^,^6^,^7^. To date, numerous methods have been developed to fabricate superhydrophobic surfaces on different substrates^8^,^9^,^10^,^11^,^12^,^13^,^14^,^15^,^16^. Recently, based on the superhydrophobic surfaces, researchers began to pay more attention on controlling and regulating the adhesion of water droplets because of potential applications such as water harvesting, biochemical analysis and lab-on-chip devices^17^,^18^,^19^,^20^,^21^,^22^. Controlling water adhesion is usually achieved through adjusting localized microstructures, chemical compositions or both of them^23^. Different technologies such as plasma treatment^24^,^25^,^26^, laser irradiation^18^,^20^,^27^,^28^, lithography^17^,^29^, ink patterning^19^,^30^,^31^,^32^, etc.^21^,^22^, have been applied to realize those adhesion controllable surfaces. For instance, Chen *et al.* achieved groove patterns on the hydrophobic polydimethylsiloxane (PDMS) surface by a femtosecond laser. The prepared surface exhibits controllable water adhesion and anisotropic wetting properties^20^. Jiang *et al.* prepared a PDMS pillars-array film by a two-beam interference laser microfabrication system. With the curvature of the film varying, the superhydrophobicity from high-adhesion state to low-adhesion state can be reversibly turned^18^. Megaridis *et al.* used localized thermal treatment to create micro adhesion controllable superhydrophilic patterns on the superhydrophobic polysilsesquioxane-silica composite coatings and when the laser was applied, the pattern scale could be down to 100 μm^28^. Milionis *et al.* fabricated a surface with controllable adhesion by spray-coating of polytetrafluoroethylene sub-micrometer particles and hydrophobically capped oxide colloidal nanoparticles on the SU-8 micro-pillar patterns. It is feasible to fabricate low-adhesion superhydrophobic surfaces with localized high-adhesion areas^17^. Based on the principles of capillary-induced adhesion, Gao *et al.* presented three types of porous nano-structured layers, which can transform between high-adhesion state and low-adhesion state^19^. Among them, the femtosecond laser can be used to make nano-scaled features on a wide range of materials such as metal, polymer and silicon^33^,^34^, however, the high cost of this technique makes it difficult to be applied for large-scale industrial productions. Lithography is another technique to make micro and nano patterns on various substrates, however, it is difficult to control substrate roughness directly, which makes it unsuitable to regulate the roughness of the patterns and further control the adhesion. Additionally, it can be seen that most of the surfaces with controllable adhesion were constructed on the polymers, such as polyaniline, SU-8 and PDMS. Metal materials have various applications in engineering and agriculture due to the strong mechanical strength and good machinability. However, to the best of our knowledge, controllable adhesion state through structure arrangement on metal surface, which is expected to have many applications in engineering, has rarely been reported.

Low-surface-energy structures with different levels of roughness exhibit different levels of adhesions^20^,^35^; an extremely rough surface morphology with low surface energy endows the surface with superhydrophobicity, while smooth or ideally flat surfaces with low surface energy would be adhesively hydrophobic or even hydrophilic. Therefore, patterns with controllable adhesion on superhydrophobic surfaces, which have important potential applications in lab-on-chip devices, could be realized through regulating the localized roughness of the surface. Electrolyte jet machining (EJM) is a mild and easy-to-implement fabrication technique based on electrochemical dissolving principle of anode. This technique could be applied to machine patterns, e.g. dimples and lines, on diverse metal matrices. In addition, patterns with different levels of roughness could be realized by adjusting the processing parameters, such as processing time and processing voltage.

Herein, a method combining electrochemical etching, EJM and surface modification was presented for the first time to construct patterns with controllable adhesion on the superhydrophobic Al alloy surfaces. Firstly, electrochemical etching was applied to fabricate micro-nano structures on the Al alloy substrate. Then, dimples with different levels of roughness were obtained on the etched surface through EJM under different processing parameters. Finally, after modification with low surface energy materials, the dimples with different roughness exhibited different levels of adhesion. Such dimples could also be fabricated on diverse metal substrates to realize localized high adhesion. The adhesion mechanism was investigated through experiments.

## Results

### Fabrication of superhydrophobic surfaces with micro dimples for droplet manipulation

**Figure 1:**
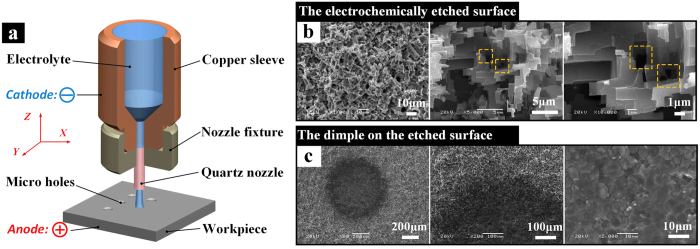
(**a**) Schematic of the process for fabricating dimples on the etched Al plate. (**b**) SEM images of the electrochemically etched surface at different magnifications. (**c**) Top-view image of the dimple, which was fabricated using the EJM technique: the rounded dimple was obtained by processing for 3 s at 100 V with a 520 μm inner diameter nozzle. The middle one is the magnified image of the boundary between the edge of the dimple and the etched rough area. The right one is the magnified image of the internal surface in the dimple.

Micro dimples with various roughness and dimensions on the electrochemically etched Al plate were fabricated using the EJM technology (Fig. 1a), which is a mild machining method. After modification with the 0.05 mol/L ethanol solution of stearic acid, the electrochemically etched areas exhibit excellent superhydrophobicity while the dimples exhibit specific adhesion to droplets due to the lack of extremely rough structures. Sphere-like droplets can be pinned to the dimples forming different patterns or slide at designed angles without residual water left.

Figure 1b shows the top-view SEM images of the electrochemically etched Al surface. Large numbers of micro holes and protrusions homogeneously covered the surface. Micro rectangular-shaped plateaus with sizes of a few microns attached to each other and therefore formed the micro- and sub-micro-scale cavities (the dashed rectangle regions in Fig. 1b) and step-like structures (Fig. 1b). The dimple was produced on the aforementioned electrochemically etched surface by the EJM technique. Figure 1c was the SEM images of the dimple obtained by processing for 3 s at 100 V with a 520 μm inner diameter nozzle. The diameter of the dimple was about 680 μm (the left one in Fig. 1c) and the boundary between the edge of the dimple and the rough etched area was distinct (the middle one in Fig. 1c). The internal surface of the dimple was smoother than the outside superhydrophobic areas, has sub-micro-scale cracks and abundant protrusion with sizes ranging from 1 to 10 μm (the right one in Fig. 1c).

**Figure 2:**
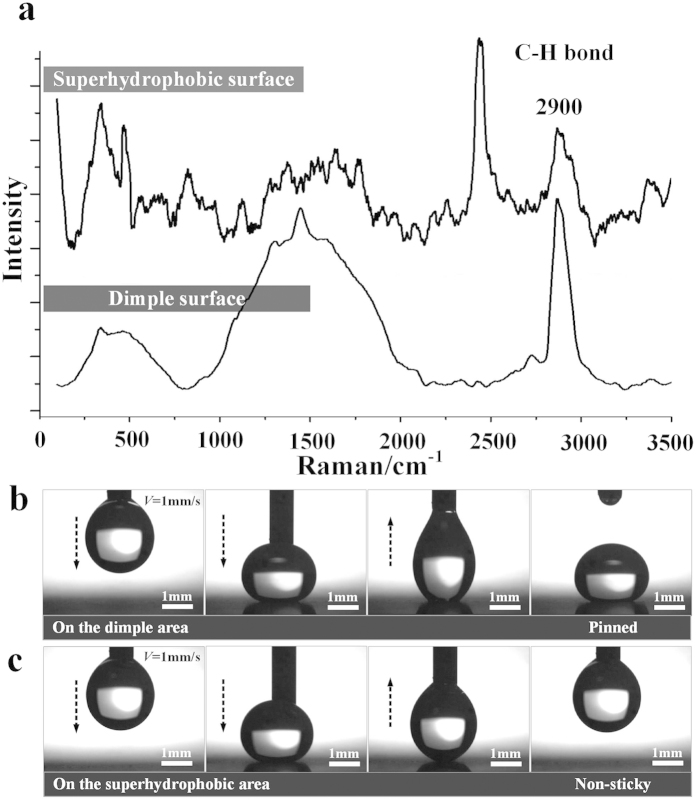
(**a**) Raman spectrum of the superhydrophobic surface and the dimple surface after modification with stearic acid. (**b**) Experimental optical images of placing a 5 μL droplet on the dimple which was processed for 3 s at 100 V with a 520 μm inner diameter nozzle. (**c**) Experimental optical images of placing a 5 μL droplet on the superhydrophobic area. The motion velocity of the needle was 1 mm/s.

Figure 2a shows the Raman spectrum of the electrochemically etched area and the dimple surface after modification with stearic acid. The characteristic peaks around 2900 cm^−1^ correspond to the C-H bond of stearic acid, which indicates the etched area and the dimple surface were all well modified by the stearic acid^36^,^37^. Then the wettability was tested with 5 μL water droplets. The CA and the sliding angle (SA) were 162.1° and 9.5° respectively on the area without EJM treatment, indicating that the Al samples prepared by electrochemical etching have excellent superhydrophobicity and the water droplet on this area is in Cassie-Baxter state^38^. However, when water droplets were laid on the dimple, there appeared additional adhesion energy. Figure 2b,c show that 5 μL water droplets respectively touched the dimple and the superhydrophobic area. The dimple was processed for 3 s at 100 V using a 520 μm inner diameter nozzle. The droplet was easily released from the needle, and pinned on the dimple when touching the dimple surface (Fig. 2b). On the contrary, the adhesion on the superhydrophobic surface was so small that the droplet just left the surface and kept sticking to the needle (Fig. 2c), indicating that the dimple has stronger adhesion energy than that of the superhydrophobic surface (See Supplementary Video S1). And a 15 μL water droplet would slide off at averaged 18.2° on that dimple, which was much higher than 5° on the superhydrophobic areas. More importantly, by adjusting the processing parameters, water droplets could slide off the dimples at designed angles without residual water left on it, which could be applied for lossless droplet manipulation.

### Dimples with controllable adhesion

**Figure 3:**
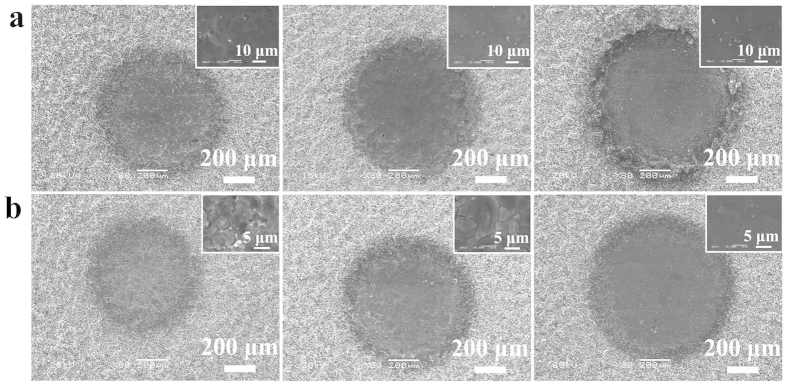
SEM images of dimples fabricated using the EJM technique. (**a**) Dimples obtained by processing for 1 s (the left), 5 s (the middle) and 10 s (the right) respectively at 300 V. (**b**) Dimples obtained by processing for 3 s at 100 V (the left), 200 V (the middle) and 400 V (the right) respectively. The inserted images are the magnified images of areas in the dimple surfaces. The inner diameter of the nozzle was 520 μm.

**Figure 4:**
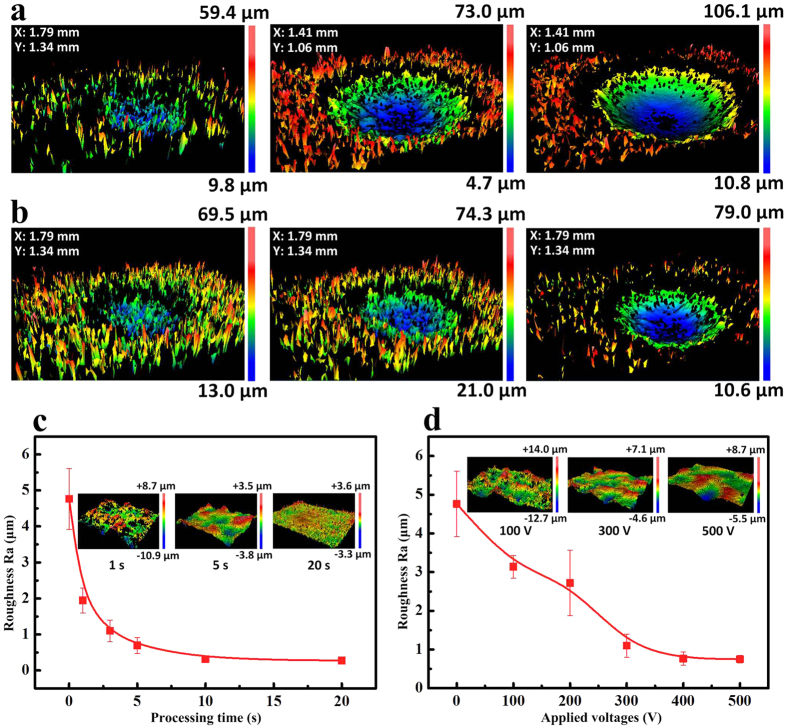
(**a**) 3D models of dimples processed for 1 s (the left), 5 s (the middle), and 10 s (the right) respectively at 300 V. (**b**) 3D models of dimples processed for 3 s at 100 V (the left), 200 V (the middle) and 400 V (the right) respectively. The inner diameter of the nozzle was 520 μm. (**c**) Roughness of the dimple surfaces fabricated for different processing time (at an applied voltage of 300 V). (**d**) Roughness of the dimple surfaces fabricated at different applied voltages (at a processing time of 3 s). The inserts in (**c**,**d**) are the 3D models of the dimple surfaces. The measurement range of the inserts in (**c**,**d**) is 0.35 mm in x-axis and 0.26 mm in y-axis.

To systematically analyze the adhesion phenomena, dimples with various profiles were produced on the superhydrophobic Al surface with different processing parameters. 7 dimples marked as Dimple 1–7 were obtained by processing for 1 s, 2 s, 3 s, 5 s, 10 s, 15 s and 20 s at a voltage of 300 V using a 520 μm inner diameter nozzle. 11 dimples marked as 8–18 were processed for 3 s at voltages ranging from 100 V to 600 V with an increase of every 50 V. As the processing time or the applied voltages increased, the surface of the dimples got smoother and the boundary between the edge of the dimple and the rough superhydrophobic area gradually become distinct (Fig. 3). 3D models (Fig. 4a,b) and surface profiles (See Supplementary Fig. S1) of the dimples measured by a 3D surface profilometer also indicate that the dimple surface was rough at first and tended to smooth with the increase of either processing time or applied voltages. As shown in Fig. 4c, the roughness of the dimple surfaces declined rapidly from Ra 4.7 μm at 0 s to Ra 0.7 μm at a processing time of 5 s, and then tended to stabilize at Ra 0.3 μm after 20 s. Figure 4d shows that the roughness decreased gradually to Ra 0.8 μm at an applied voltage of 400 V. This roughness variation with processing parameters also indicates that the EJM is a feasible technique to regulate the roughness of patterns on superhydrophobic surfaces.

**Figure 5:**
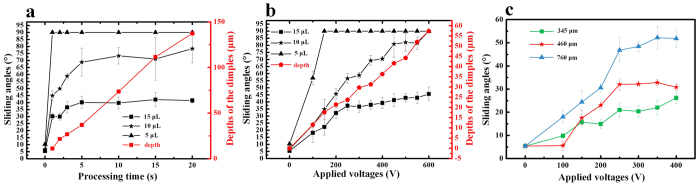
The SAs of water droplets with different volumes on different dimples. (**a**) Depths of the dimples and SAs of different volume water droplets on dimples processed for different time. The diameter of the nozzle was 520 μm and the applied voltage was 300 V. (**b**) Depths of the dimples and SAs of different volume water droplets on dimples processed at different voltages. The diameter of the nozzle was 520 μm and the processing time was 3 s. (**c**) The SAs of 15 μL droplets on dimples processed at different voltages with different nozzles. SAs of 90° mean that the droplets were pinned and could not slide off.

SAs on Dimple 1–7 firstly increased with the increase of the processing time and then began to stabilize around 5 s while depths of the dimples increased linearly with the increasing processing time (Fig. 5a). Analogously, SAs on Dimple 8–18 increased with the increase of the applied voltages and tended to be steady after 250 V in spite of the linear increase of the depths of the dimples (Fig. 5b). And SAs of 15 μL droplets on the dimples fabricated by nozzles with inner diameters of 345 μm, 460 μm and 760 μm had the similar trend as (Fig. 5c).

## Discussion

**Figure 6:**
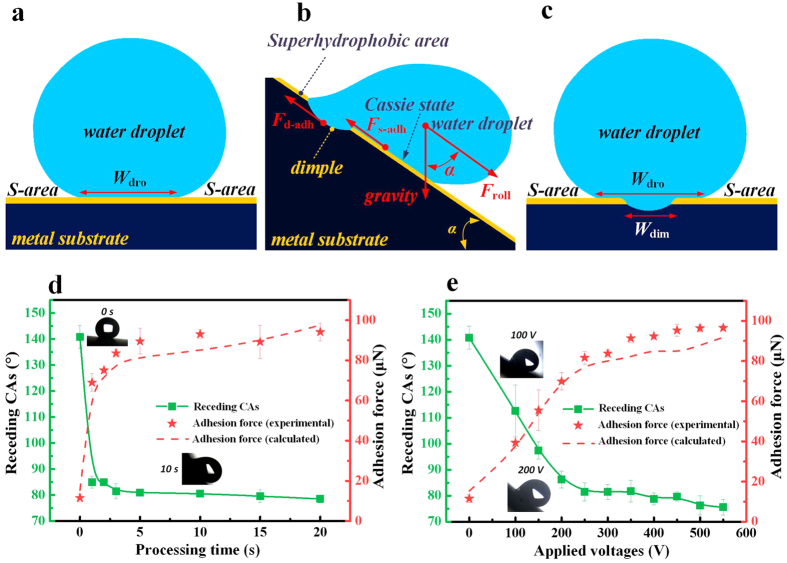
(**a**) Schematic illustration of the interfacial area of water droplet on the superhydrophobic area. (**b**) Schematic illustration of the adhesion mechanism. (**c**) Schematic illustration of the interfacial area of water droplet on the dimple. Plot of the receding CAs and the adhesion force of 10 μL droplets on the dimples that were fabricated (**d**) for different time (at an applied voltage of 300 V) and (**e**) at different applied voltages (at a processing time of 3 s).

Based on the studies in the mid-twentieth century^39^^–^41, the adhesion force (*F*_adh_) that restricts the droplet from sliding off an inclined surface is proportional to the width (*W*_dro_) of the droplet-solid interface (Fig. 6a) that is perpendicular to the sliding direction. This can be expressed as the following semi-empirical Furmidge equation^40^:


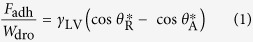


where *γ*_LV_ is the liquid surface tension, 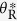 and 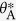 are the receding and advancing CA of the droplet on the surface. When the droplet was laid on the dimple area, the droplet-substrate interface turned into composite, that is, besides the *F*_s-adh_ between the droplet and superhydrophobic area (both advancing and receding CA of the etched superhydrophobic Al surface), there would be an additional contribution which is the adhesion force *F*_d-adh_ between the droplet and dimple surface (advancing CA of the superhydrophobic surface and receding CA of the dimple surface) to the overall adhesion force *F*_overall_ (Fig. 6b). Assuming these two adhesion force is independent, the predicted adhesion force *F*_overall_ that prevents the droplet from sliding away from a dimple can thereby be calculated as the following equation^31^:


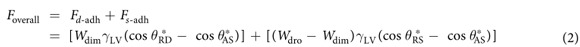


where *W*_dim_ is the diameter of the fabricated dimple (Fig. 6c), 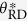 is the receding CA of the droplet on the dimple, which can be experimentally measured. 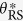 and 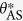 are the receding and advancing CA of the droplet on the electrochemically etched superhydrophobic surface, and *W*_dro_ is the overall droplet-substrate interfacial width, which can also be obtained by experimental measurements. Based on equation (2), it can be inferred that different adhesion force acting on the droplets could be realized by regulating the 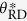 or the dimple diameter *W*_dim_. As illustrated in Fig. 6d,e, the increase of either the processing time or the applied voltages would result in the decrease of 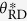, showing that the EJM technique can be applied to adjust the localized 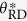 on a superhydrophobic surface. The adhesion force that prevents the droplets from sliding off can therefore be calculated based on equation (2) using the measured 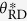, *W*_dim_ and *W*_dro_. The results shown in Fig. 6d,e demonstrate that the calculated values are in good agreement with the experimental values.

**Figure 7:**
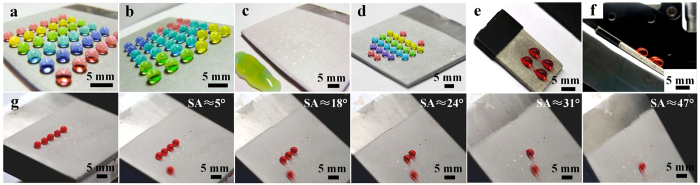
Examples of adhesion-controlling patterns on superhydrophobic surface. (**a**) Water droplets of 20 μL stayed firmly on the surface patterned by the 6 × 6 dimples, which were processed for 5 s each at 300 V with a 520 μm inner diameter nozzle and assisted by a three-axis motion system. (**b**) Water droplets kept pinned on that tilted patterned surface. (**c**) Water droplets all slid off the surface at a tilted angle of about 41°. (**d**) Heart-shaped patterned water droplets on the surface. (**e**) 20 μL water droplets were pinned by the 2 × 2 dimples on a superhydrophobic magnesium surface. (**f**) Side view of (**e**). (**g**) Droplets slid at designed angles at appointed positions.

In order to explore the potential applications of the dimples, 6 × 6 arrayed dimples processed for 5 s each at a voltage of 300 V with a 520 μm inner diameter nozzle were obtained on the superhydrophobic Al alloy surface (Fig. 7). The spacing between the dimples was 5 mm. The water droplets with volumes of 20 μL can stay steadily on the patterned surface (Fig. 7a) and would not slide off even the surface was tilted (Fig. 7b). When the surface was tilted at about 41°, all the water droplets would slide off (Fig. 7c). Figure 7d shows a heart-shaped pattern formed by the droplets on the arrayed dimples. The EJM technique follows the principle of anodic dissolution, so it can be extended to fabricate patterns on various metal matrixes. Figure 7e,f show a 2 × 2 dimple array with 5 mm spacing obtained on a superhydrophobic magnesium surface and 20 μL water droplets were pinned firmly on the tilted surface. By fabricating dimples with different parameters at specified positions, droplets could slide at designed angles with or without residual water left at appointed positions (see supplementary Video S2). Left to right, five 15 μL droplets on superhydrophobic area and on the four dimples slid at about 5°, 18°, 24°, 31° and 47° respectively (Fig. 7g). The four dimples on the superhydrophobic Al alloy surface were processed for 3 s at 100 V, 150 V, 200 V and 250 V with a 760 μm inner diameter nozzle.

For the EJM technique used in the experiment, the minimal dimple size depends on the inner diameters of the nozzles while the minimal distance between the dimples is closely related to the minimal dimple size and the precision of the motion platform. Therefore, technically, dimples with diameters of several tens of microns can be fabricated on various metal materials and the spacing between the dimples can also achieve the level of hundreds of microns. Supplementary Fig. S2 shows the minimal dimple array that was fabricated using our set-up EJM unit. The average diameter of the fabricated dimples is 150 μm and the distance between dimples is about 290 μm. Compared with laser, the EJM does suffer defects such as limited machinable materials and relatively low machining precision. However, the EJM unit is much cheaper and easier to construct, which makes it more suitable for large scale industrial productions.

In conclusion, electrolyte jet machining (EJM) was applied for the first time in constructing dimples with different levels of adhesion by regulating the processing parameters. The surface fabricated by EJM gradually smoothed with the increase of processing time and voltage. By adjusting the processing parameters, dimples with different levels of roughness and adhesion could be obtained at specified positions. Water droplets with different sizes may be firmly pinned to the surface forming various patterns and could slide off at designed angles at appointed positions. Based on this adhesion mechanism, this method could be applied in constructing various patterns with controllable adhesion on diverse metal matrixes. On account of these specific adhesion phenomena, this research can be applied for biological and optical fields such as trace analysis of aqueous solution, water harvesting and sensors.

## Methods

### Materials

6061 aluminum alloys plates (3 mm thick) were purchased from Suzhou Metal Material Manufacturer (China). Stearic acid [STA, CH_3_ (CH_2_)_16_COOH], NaCl and NaNO_3_ were purchased from Tianjin Kermel Chemical Reagent Co. (China). All chemicals were analytical grade reagents and were used as received.

### Fabrication of micro-nano structures

The Al alloys samples (60 mm × 60 mm) were polished using #800 and #1500 abrasive papers, and ultrasonically cleaned with deionized water to remove the oxide layer, grease and other impurities. The cleaned Al plate was then electrochemically etched at a current density of 600 mA·cm^−2^ for 8 min in the 0.1 mol·L^−1^ NaCl aqueous solution, and subsequently ultrasonically rinsed with water to clear away the impurity particles on the surface.

### Fabrication of superhydrophobic surfaces with micro dimples

The dimple patterns with various profiles on the etched Al plate were obtained using the EJM technique. This EJM machining system mainly consists of copper-sleeve cathode, nozzle fixture, electrolyte, and quartz nozzle. The quartz nozzle is fixed on the nozzle fixture with sealant while the nozzle fixture is fixed on the copper sleeve through thread. When the cathodic copper sleeve and the anodic substrate was connected with high voltage DC power, the cyclic electrolyte would be charged negatively when flowing through the copper sleeve, subsequently shoot on the substrate and remove materials based on the electrochemical dissolving principle of anode. The glow discharge will occur in the EJM process when a high voltage is applied. Hence, the EJM process is a synthetic process that consists of electrochemical machining, chemical machining and electrical discharge machining process^42^. The electrolyte flows outward radially when impacting the workpiece surface. The hydraulic jump occurs at a certain distance from the center of the jet flow. Therefore, the current is concentrated in this region and materials could be removed selectively^43^^–^45.

15 wt% NaNO_3_ aqueous solution was used as the electrolyte in the EJM process. The electrolyte pressure was 0.3 MPa. The interelectrode gap, that is, the distance between the tail end of quartz nozzle and the Al plate surface, is 1 mm. Dimples with different profiles were fabricated on the electrochemically etched Al plate under different processing parameters. After this process, the Al plate with these dimples was ultrasonically cleaned in the deionized water, subsequently, dried and immersed in 0.05 mol/L ethanol solution with stearic acid [CH_3_(CH_2_)_16_COOH] for 30 min to lower the surface energy, and then a superhydrophobic Al alloy plate with specific dimples was obtained.

### Characterization

The morphology of the samples was characterized by a scanning electron microscope (SEM, JSM-6360LV, Japan). The 3D models and surface profiles of samples were characterized by a 3D surface profilometer (ZYGO NewView 5022 3D optical surface profiler). Optical images of water droplets were captured by a CCD camera (Microvision MV-VD030SC, China). Contact angle is defined as the angle between the solid-liquid interface and the tangent of liquid-vapor interface at the contact point of solid-liquid-vapor^46^, and measured using a mapping program based on the definition at ambient temperature. Sliding angle is defined as the angle at which the water droplet begins to roll off the gradually inclined surface^47^ and was measured by a precision whirler.

## Additional Information

**How to cite this article**: Yang, X. *et al.* Controlling the Adhesion of Superhydrophobic Surfaces Using Electrolyte Jet Machining Techniques. *Sci. Rep.* **6**, 23985; doi: 10.1038/srep23985 (2016).

## Supplementary Material

Supplementary Information

Supplementary Video S1

Supplementary Video S2
