# Supplementary material for: Controlling the Adhesion of Superhydrophobic Surfaces Using Electrolyte Jet Machining Techniques
Source: Sci Rep. 2016 Apr 5;6:23985. doi: 10.1038/srep23985 (PMC4820749; doi:10.1038/srep23985)
Supplement: Supplementary Information [file srep23985-s1.pdf]

Supplementary Information

Controlling the Adhesion of Superhydrophobic Surfaces Using Electrolyte Jet Machining Techniques

Xiaolong Yang[1], Xin Liu[1], Yao Lu[2], Shining Zhou[1], Mingqian Gao[1], Jinlong Song[1,*] & Wenji Xu[1]

[1] Key Laboratory for Precision and Non-traditional Machining Technology of the Ministry of Education, Dalian University of Technology, Dalian 116023, People's Republic of China.

[2] Department of Chemistry, University College London, 20 Gordon Street, London, WC1H 0AJ, UK.

*To whom correspondence should be address: E-mail: *songjinlong@dlut.edu.cn*

Supplementary Videos

Video S1: Video of high-water-adhesion phenomenon on the dimple. When water droplets were laid on the dimple, there appeared additional adhesion energy. Video S1 shows that 5 μL water droplets respectively touched the superhydrophobic area and the dimple which was processed for 3 s at 100 V with a 520 μm inner diameter nozzle. The droplet was easily released from the needle, and pinned on the dimple when touching the dimple surface. On the contrary, the adhesion on the superhydrophobic surface was so small that the droplet just left the surface and kept sticking to the needle, indicating that the dimple has stronger adhesion energy than that of the superhydrophobic surface.

Video S2: Video of water droplets sliding at designed angles at appointed positions. By fabricating dimples with different parameters at specified positions, droplets could slide at designed angles with or without residual water left at appointed positions. Left to right in the Video, five 15 μL droplets on superhydrophobic area and on the four dimples slid at about 5°, 18°, 24°, 31° and 47° respectively. Left to right, the four dimples on the superhydrophobic Al alloy surface were processed for 3 s at 100 V, 150 V, 200 V and 250 V using a 760 μm inner diameter nozzle.

Supplementary Figures

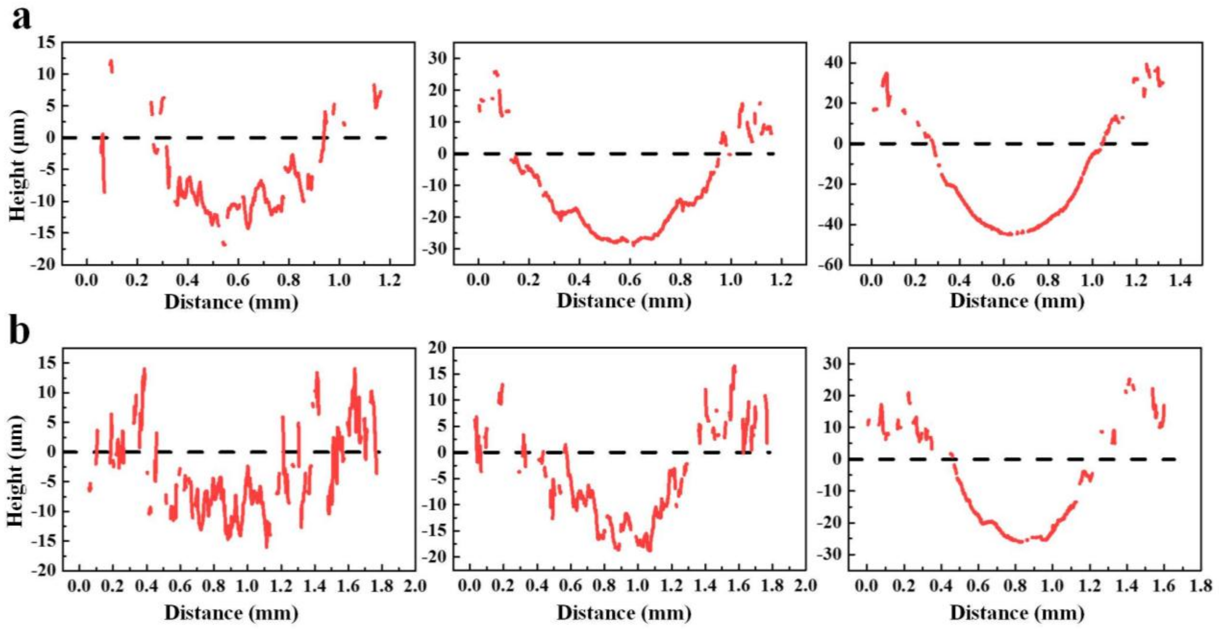

Supplementary Figure S1. (a) Profiles of dimples processed for 1 s (the left), 5 s (the middle), and 10 s (the right) respectively at 300 V. (b) Profiles of dimples processed for 3 s at 100 V (the left), 200 V (the middle) and 400 V (the right) respectively. The inner diameter of the nozzle was 520 μm.

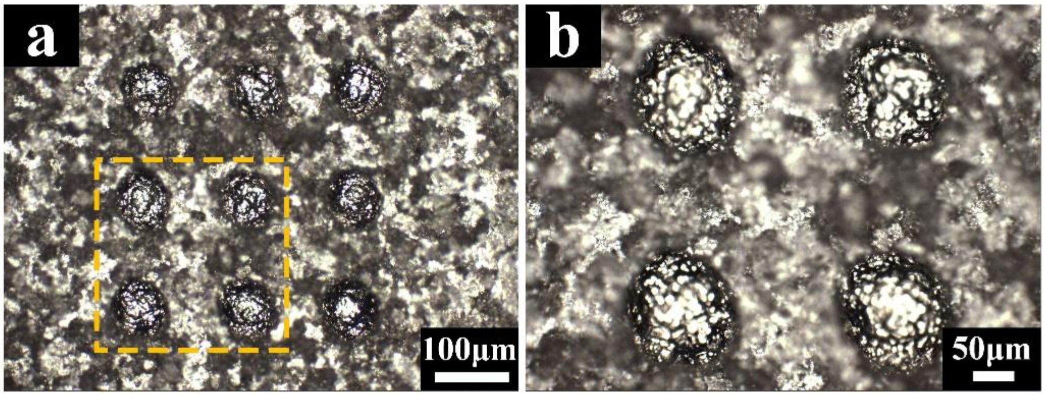

Supplementary Figure S2. (a) Microphoto of 3×3 dimple array fabricated by our EJM unit using a nozzle with an inner diameter of 100 μm. The applied voltage was 300 V and the processing time was 10 s. (b) Magnified images of the yellow rectangle area in (a). The average diameter of the dimples is 150 μm and the distance between dimples is about 290 μm.
